# Ethics knowledge, attitudes, and experiences of tertiary care pediatricians in Ethiopia

**DOI:** 10.1186/s12910-022-00812-w

**Published:** 2022-07-22

**Authors:** Atnafu Mekonnen Tekleab, John D. Lantos

**Affiliations:** 1 Department of Pediatrics and Child Health, St Paul’s Hospital Millennium Medical College, Addis Ababa, Ethiopia; 2 JDL Consulting, Ossining, NY USA

**Keywords:** Bioethics, Pediatrics, Developing country

## Abstract

**Background:**

Pediatricians in developing countries face different ethical dilemmas than do doctors working in settings with more resources. There are very few studies from developing countries analyzing pediatricians’ knowledge and attitudes regarding the ethical dilemmas that arise in such settings. To address this gap, we explored the clinical ethical knowledge, attitude and experience of physicians who are working in the Department of Pediatrics and Child Health (DPCH) of St Paul’s Hospital Millennium Medical College (SPHMMC), Addis Ababa, Ethiopia.

**Study population:**

All pediatric resident doctors and pediatric consultants who were working in the DPCH of SPHMMC in December, 2020.

**Method:**

A structured pretested self-administered questionnaire was distributed to all 79 of the residents and consultants in the department during the period December 15–27, 2020. The questionnaire assessed the knowledge (23 questions), attitude (9 questions) and experiences (9 questions) of the study participants regarding a variety of bioethical issues. Data were analyzed using SPSS version 20.0 for windows. The mean, median, standard deviation, and interquartile range of respondents’ scores were determined and compared using Fisher’s exact test.

**Result:**

A total of 59/79 (75%) physicians completed the questionnaire. The mean age of the participants was 30.7 ± 4.1 years. Thirty six (61.0%) were female. At the time of data collection, more than half (57.6%) served < 5 years as a physician. The mean ethics knowledge score of the respondents was 12.3 ± 2.34 out of 23 knowledge questions. The lowest and highest knowledge scores were 8 and 19 respectively. Scores were highest on questions about confidentiality (94.9% correct) and lowest on questions about genetic testing and diagnosis (13.6% correct). Only 13 (22.4%) physicians agreed with the practice of children should never be treated without consent of the parent.

**Conclusion:**

Tertiary care pediatricians at one hospital in Ethiopia lack knowledge about current standards in bioethics. There is a need for more ethics education in this setting.

## Background

Ethical dilemmas arise frequently in the practice of pediatrics. Practitioners must analyze moral principles and values in order to make clinical judgments about what is best for patients and families [1] and thus improve the quality of patient care. Knowledge of clinical ethics can help doctors to identify, analyze and resolve ethical problems that arise during practice [2].

In high-income countries, hospital ethics committees (HECs) help practitioners deal with ethical challenges that arise during patient care. These problems become ever more complex as technology allows more and more children with complex chronic illnesses to survive. Many such children require frequent admissions to intensive care units and frequent discussions about the wisdom of continuing life-support [3]. Dealing with such patients poses ethical challenges. Pediatricians must balance considerations of beneficence, autonomy, non-maleficence and justice. They must recognize that, while young children lack autonomy, teens are developing it. They must address ethical dilemmas such as validity of consent during patient management, providing futile care, resource constraint, and the extent of patient involvement in decision making [4] Practitioners must decide when and how to include the child’s voice in the process of shared decision making [5]. Therefore, it is important to educate doctors about prevailing ethical guidelines [6].

Professional education is one crucial function of HECs. Few such committees exist in developing countries [7]. In the absence of trained ethics consultants, front-line clinicians must address the ethical challenges that arise during patient care. We sought to understand the knowledge, attitudes, and experiences of these doctors regarding pediatrics bioethics. We hope that the results will help plan training at our hospital and similar hospitals in developing countries.

## Methods

### Study setting

The study was conducted in SPHMMC, Addis Ababa, Ethiopia. The hospital is a teaching and referral hospital that provides tertiary level acute clinical care. The hospital has 150 pediatric beds with about 30,000 pediatric patients visiting the hospital per year. At the time of data collection, the Department of Pediatrics and Child Health had 16 consultants and 63 pediatric residents. A consultant is faculty member of the department who is a certified pediatrician or certified in any of pediatric sub-specialties. Of these 79 physicians, two had prior ethics training. There was no standing clinical ethics committee in the DPCH at the time of the data collection.

### Data collection

Data were collected using a structured pretested self-administered questionnaire. The questionnaire was pretested by administering it to pediatricians who were working in another teaching hospital in the city. Based on their feedback, we did minor modification to the questionnaire and distributed it to the 79 pediatricians at SPHMMC during the period of December 15–27, 2020.

The questionnaire was designed to address the following characteristics of the respondents: background characteristics, knowledge on certain domains of pediatric bioethics, attitude and experiences of ethical dilemmas that the clinicians encountered during their practice. It enquired on the knowledge of respondents on the following domains of pediatric bioethics by administering a true–false question: protecting confidentiality, pediatric consent, genetic testing and diagnosis, neonatology, end-of-life decision and decision making for minors. Each correct response was given a score of 1 and a wrong response a score of 0. Total points to be scored were 23 and the minimum was 0. The mean and its standard deviation (sd) of the knowledge score was determined. A score above 19 was considered as good knowledge [8].

The questionnaire was used after obtaining permission from the authors who had designed it [8].

The attitude and experience sections of the questionnaire were developed based on information obtained through literature review. Attitude was assessed using Likert scale responses to 9 questions. The scoring system ranged from strongly disagree 1, disagree 2, neutral 3, agree 4, and strongly agree 5. The responses were summed up and a total score was obtained for each respondent. The mean score was calculated and those who scored above or equal to the mean score had positive attitude and scores below the mean were considered as negative attitude towards pediatric bioethics. The highest score was expected to be 45 and the lowest score to be 9. The practice was assessed by asking the physicians’ experience towards specific vignette of ethical dilemma.

### Statistical analysis

Data were analyzed using Statistical Package for Social Sciences (SPSS) version 20.0 for windows software. Mean with is standard deviation and median with its Interquartile range were determined when necessary. Descriptive data were presented in text form and by using frequency tables. Fischer’s exact test was used when appropriate and statistical significance was considered when p-value was less than 0.05.

## Result

### Background characteristics of the physicians

A total of 59/79 (75.0%) physicians completed the questionnaire (12 consultants, 19 year-1 pediatric residents, 9 year-2 pediatric residents and 19 year-3 pediatric residents). The mean age of the participants was 30.7 ± 4.1 years. Thirty-six (61.0) were female. Most 53/59 (89.8%) were Christian by religion and about half 30/59 (50.8%) had at least one child of their own. At the time of data collection, more than half (57.6%) served < 5 years in clinical practice. Four physicians’ practice was confined to single service area (NICU-3 physicians and PICU-1 physician) while the rest were rotating at different service units in the department (emergency, NICU, PICU and chronic follow up clinics) (Table 1). Only two consultants had prior pediatrics bioethics training.

**Table 1:** Background characteristics of the study participants (n = 59), 2021

Characteristics	Number (%)
*Age*	
26–29	27 (45.8)
30–33	22 (37.3)
34–37	5 (8.5)
38–41	4 (6.8)
> 41	1 (1.7)
*Sex*	
Male	23 (39.0)
Female	36 (61.0)
Marital status	
Married	32(54.2)
Single	26 (44.1)
Divorced	1 (1.7)
*Do you have child?*	
Yes	30 (50.8)
No	29 (49.2)
*Year of service as physician*	
3–5	34 (57.6)
6–8	14 (23.7)
> 8	11 (18.6)
*Academic status*	
Consultants	12 (20.4)
Year I residents	19 (32.2)
Year II residents	9 (15.3)
Year III residents	19 (32.2)
*Service area of assignment*	
Limited to PICU	1 (1.7)
Limited to NICU	3 (5.1)
Rotating to ward, ER^*^, NICU^**^, PICU^*#^, follow up clinics	55 (93.2)

^*^ER Emergency department, ^**^NICU neonatal intensive care unit, ^*#^*PICU* pediatric intensive care unit

### Pediatric bioethics knowledge status of the respondents

Using true/false questions, we assessed physicians’ knowledge of the following domains of pediatric bioethics: protecting confidentiality, pediatric consent, genetic testing and diagnosis, neonatology, end-of-life decision and decision making for minors. The mean and median knowledge scores of the respondents were 12.3 ± 2.34 and 12.0 (IQR-10,14) respectively. Slightly over half (30/59) scored less than the mean knowledge score. The lowest score was 8 and it was scored by two respondents (year 1 and year 3 residents) while the highest score was 19 and it was scored by year 3 resident.

Results of knowledge assessments are shown in Table 2. The most correctly answered domain of knowledge question was regarding protecting confidentiality. 95% of respondents answered it correctly. The least correctly answered question was regarding genetic testing and diagnosis. Only 14% of respondents answered it correctly. The statements regarding consenting a mature minor and when to consider an emancipating minor were correctly answered by 27/59 (45.8%) and 43/59 (72.9%) physicians respectively.

**Table 2:** Performance of the respondents towards the ethics knowledge questions (n = 59), 2021

Knowledge questions	Proportion of physicians who answered correctly N (%)
*Regarding the decision to resuscitate at threshold of viability*	
•The decision to resuscitate should alter in the delivery room and in the perinatal period if the neonates condition at birth is much different than was expected prenatally	42 (71.2)
•The decision to resuscitate when born at the threshold of viability has to involve consultation with the hospital legal team	9 (32.2)
•The decision to resuscitate when born at the threshold of viability should generally involve consultation with colleagues	17 (28.8)
*Regarding Withholding and withdrawing fluids and nutrition*	
•Withholding and withdrawing of medically provided fluids and nutrition can be done for the same reasons	29 (49.2)
•There is fundamental ethical distinction between deciding not to start a life sustaining treatment and deciding to stop a life sustaining treatment that has already been started	10 (17.0)
*Regarding Life sustaining treatment*	
•Mature minor’s refusal for further life sustaining medical treatment ought to be respected	16 (27.1)
•Provide comfort by giving large doses of analgesics and sedatives even if they cause the patient to become obtunded	24 (40.7)
•Enteral nutrition can be ethically withdrawn from a patient who is in a vegetative state	13 (22.0)
•The physician is ethically justified to provide care even if the parents of 4 month old infant refuse to consent to vaccinate their child	19 (32.2)

Residents in year 3 were most likely to have accurate knowledge about end-of-life decisions than were other physicians. The difference between year 3 residents and all other physicians regarding end-of-life decisions was statistically significant (*P* = 0.007).

### Attitude of the respondents towards end-of-life care

Questions about attitudes revealed differences in doctors’ values with regard to decisions about withholding or withdrawing life-support. For example, we asked which considerations would guide their decisions about life-support for a 12 year old in persistent vegetative state. The vast majority 49/58(85%) would consider the family’s assessment of the patient’s quality of life. Three quarters considered cost to society and nearly half considered the risk of litigation. Overall results are shown in Table 3.

**Table 3:** Factors influencing the physicians’ attitude towards the care of a hypothetical patient who is in vegetative state (n = 58), 2021

Factor influencing the extent of care	Proportion of physicians who said the stated factor is “*important or very important*” in influencing their decision towards the extent of care provided, n (%)
Quality of life as viewed by the patient	42 (72.4)
Quality of life as viewed by the family	49 (84.5)
Patient unlikely to survive	39 (67.2)
Fear of litigation or breaking the law	28 (48.3)
Financial cost to the society	44 (75.9)
Intensive Care Unit bed availability	47 (81.0)

We also found variation in physicians’ willingness to give narcotics or anxiolytics to dying patients. Of the 57 respondents, 33/57 (57.9%) physicians said “it is important/very important” for them to add or increase the dose of narcotics and 23/57 (40.4%) physicians said it is “important/very important” to add or increase the dose of benzodiazepines to comfort the patient (Table 4).

**Table 4:** Physicians’ perception towards adding/increasing narcotics/benzodiazepines dose to comfort patient in persistent vegetative state, (n = 57), 2021

Attitude question	Physicians’ who perceived the action as important/very important, n (%)
Adding or increasing narcotics dose to comfort patient in PVS^*^	33 (57.9%)
Adding or increasing benzodiazepines dose to comfort patient in PVS	23 (40.4%)
Disclosing the medical error after two doses of phenobarbitone were wrongly administered to the patient	36 (63.2%)

^*^PVS Persistent vegetative state

### Attitudes about specific ethical practices

The practice of ethics by the respondents was assessed by asking the extent of their agreement with the practice of respecting patient autonomy, disclosing medical error, confidentiality, consent, physician paternalism and informing patient condition to close relatives. Table 5 shows great variation in respondents’ values regarding these controversial issues. Only half would respect the wishes of a mature minor. 40% would not disclose a medical error to the patient.

**Table 5:** Physicians’ experience to set of ethics domains (n = 58), 2021

Practice question	Extent of physician’s agreement with the practice
	Agree/strongly agree
A mature minor’s wish must always be respected	28 (48.3%)
Medical error, if any has to be told to the patient	35 (60.4%)
Confidentiality is important	54 (93.1%)
Doctors’ should make the decision not the patient	6 (10.3%)
Consent only for surgery-not for tests and medications	1 (1.7%)
Close relatives should always be told about patient condition	6 (10.3%)
Children should never be treated without consent of parent	13 (22.4%)
Doctors should refuse to treat a violent patient	1 (1.7%)
Ethical conduct is only important to avoid legal action	6 (10.3%)

## Discussion

This preliminary study explored the ethics knowledge, attitudes, and experiences of physicians in a referral children’s hospital in Addis Ababa, Ethiopia- a resource-limited setting. We used a questionnaire that had been developed and tested in the United States. Our findings indicated that the respondents had poor knowledge of many important ethical principles. The mean knowledge score of the respondents (12.3) was lower than that reported for pediatricians in the United States where the mean knowledge score was 17.3 [8]. Scores in our survey were similar to those found among pediatricians in Nepal [9]. We found wide variation in attitudes and practices in some of the domains of pediatrics bioethics. Many doctors endorsed practices that are not recommended by practice guidelines [10–14]. For example, the American Academy of Pediatrics (AAP) states that withdrawing and withholding life sustaining Medical Treatment are ethically and morally equivalent [10, 11]. Many of the doctors in our hospital saw ethical differences between withholding versus withdrawing. When respondents were asked whether there is fundamental ethical distinction between deciding not to start a Life-Sustaining Medical Treatment and deciding to stop a life sustaining treatment that has already been started, only 10/59 (17.0%) of them responded as per the guideline recommendation.

The AAP guideline highlights that a subset of adolescents can consent for or refuse treatment [12]. However, in our survey, only 16/59(27.1%) physicians correctly agreed with the statement that “a mature minor’s refusal for further life sustaining medical treatment ought to be respected,” indicating the respondents’ knowledge gap on the subject matter. Only 22% of physicians agreed that “enteral nutrition can be ethically withdrawn from a patient who is in a persistent vegetative state.” The AAP states that it is ethical to forgo medically administered nutrition and hydration as long as they do not provide net benefit to the child and are not in the best interest of the child [10, 11, 13]. Fewer than half of our respondents agreed that it is appropriate to give a dying child large doses of analgesics or sedatives even if they cause the patient to become obtunded [14].

It is worrisome to see only 63.2% of physicians felt it is “important or very important” to disclose medical error to close relatives. Current recommendations are to disclose errors in a clear statement by detailing the error and how it will affect the health of the patient [15].

Interestingly, the highest scores in our study were among third year residents. This suggests that modern bioethical principles may conflict with traditional practices in our country. It also suggests that education is likely to be effective in changing knowledge, beliefs and attitudes. Limitations of the current study includes: small sample size of study participants and lack of adequate power for generalizability; the study was being limited to single center and hence may lack representativeness.

## Conclusion

Pediatricians practicing in a tertiary care center in Ethiopia need ethical guidance in order to care for critically ill patients while upholding the highest ethical standards. Pediatrics bioethics training should be tailored to the practice setting and must address the traditional values that have guided decisions in the past. Doctors in such settings struggle to use new and scarce life-support technology in ways that are beneficent and just. A HEC that is capable of providing consultation, education, and case discussions could help doctors understand the principles that have evolved to guide decisions in ethically complex situations.

## Data Availability

The datasets used and/or analysed during the current study are available from the corresponding author on reasonable request.

## Abbreviations

AAP: American academy of pediatrics
HEC: Hospital ethics committee
NICU: Neonatal intensive care unit
PICU: Pediatric intensive care unit
SPHMMC: ST Paul’s hospital millennium medical college
IRB: Institutional review board
SPSS: Statistical package for social studies
